# Do self-management interventions improve self-efficacy and health-related quality of life after stroke? A systematic review

**DOI:** 10.1177/17474930251340286

**Published:** 2025-04-24

**Authors:** Elizabeth A Lynch, Katie Nesbitt, Aarti Gulyani, Raymond J Chan, Niranjan Bidargaddi, Dominique A Cadilhac, Billie Bonevski, Fiona Jones, Liam P Allan, Erin Godecke, Rebecca Barnden, Emily Brogan, Thoshenthri Kandasamy, Stacy Larcombe, Lemma N Bulto, Coralie English

**Affiliations:** 1 Caring Futures Institute, College of Nursing and Health Sciences, Flinders University, Adelaide, SA, Australia; 2 Flinders Digital Health Research Centre, College of Medicine and Public Health, Flinders University, Adelaide, SA, Australia; 3 Stroke and Ageing Research, Department of Medicine, School of Clinical Sciences, Monash University, Clayton, VIC, Australia; 4 Stroke Theme, Public Health and Health Services Research Group, The Florey, Heidelberg, VIC, Australia; 5 Flinders Health and Medical Research Institute, College of Medicine and Public Health, Flinders University, Adelaide, SA, Australia; 6 Population Health Research Institute, St George’s School of Health and Medical Sciences, King’s College London, London, UK; 7 Australian e-Health Research Centre, Commonwealth Scientific and Industrial Research Organisation, Perth, WA, Australia; 8 Stroke Recovery and Rehabilitation, Perron Institute for Neurological and Translational Science, Perth, WA, Australia; 9 Allied Health Research, Sir Charles Gairdner Osborne Park Health Care Group, Nedlands, WA, Australia; 10 Peninsula Clinical School, School of Translational Medicine, Monash University, Melbourne, VIC, Australia; 11 Peninsula Health, Frankston, VIC, Australia; 12 National Centre for Healthy Ageing, Monash University, Melbourne, VIC, Australia; 13 Faculty of Health Sciences, Curtin University, Perth, WA, Australia; 14 School of Health Sciences, University of Newcastle, Callaghan, NSW, Australia; 15 Heart and Stroke Program, Hunter Medical Research Institute, New Lambton Heights, NSW, Australia; 16 Centre of Research Excellence to Accelerate Stroke Trial Innovation and Translation, University of Sydney, Sydney, NSW, Australia

**Keywords:** Stroke, self-management, self-efficacy, health-related quality of life

## Abstract

**Introduction:**

Self-management interventions are recommended after stroke in many international guidelines to improve health-related quality of life (HRQoL). Self-efficacy, a person’s confidence in their abilities, is widely considered to underpin individuals’ abilities to self-manage their health.

**Aims:**

To synthesize evidence on the effectiveness of self-management programs for improving self-efficacy or HRQoL in stroke survivors.

**Summary of Review:**

The protocol was registered with the International Prospective Register of Systematic Reviews (CRD42023440168). We searched databases including Medline, Emcare, Scopus, Cochrane Library, CINAHL, and trial registries from inception to 13/12/2024. Only randomized controlled trials (RCTs) comparing the effect of a self-management intervention to no/another intervention for survivors of stroke on self-efficacy or HRQoL were included. Risk of bias was assessed using the Cochrane Collaboration criteria. Meta-analyses for self-efficacy and HRQoL were performed using random effect model. From 13,608 abstracts screened, 44 randomized controlled trials involving 5931 participants were included. Median time post-stroke of recruited participants ranged from 14 days to 3 years. Time required to deliver the interventions ranged from 45 min to 72 h. Self-management interventions in all included trials had multiple components, predominantly education (N = 40, 91%) and goal setting (N = 39, 89%). Interventions were delivered to individual survivors of stroke (N = 18, 41%), groups of survivors (N = 15, 34%), both individual and group delivery to survivors (N = 5, 11%) and individually to survivor-carer dyads (N = 6, 14%). Interventions were delivered entirely face-to-face (N = 28, 64%), entirely by phone or video-conferencing (N = 7, 16%) or a combination of these delivery modes (N = 9, 20%). There was low certainty evidence that self-management programs compared to no intervention did not significantly improve self-efficacy on pooled effect sizes (SMD 0.08, 95%CI -0.02 to 0.18). There was moderate certainty evidence that self-management programs had a marginal significant effect on HRQoL (SMD 0.07, 95% CI 0.01 to 0.13). Limitations to the review include marked variation between included studies in the interventions delivered, and outcome measures used, targeted behaviors and time since stroke.

**Conclusion:**

Self-management programs varied markedly in content and dose. There is low-certainty evidence that currently designed self-management programs do not significantly improve self-efficacy. There is moderate certainty evidence that self-management programs have a small effect on HRQoL after stroke.

## Graphical abstract

**Graphical abstract:**
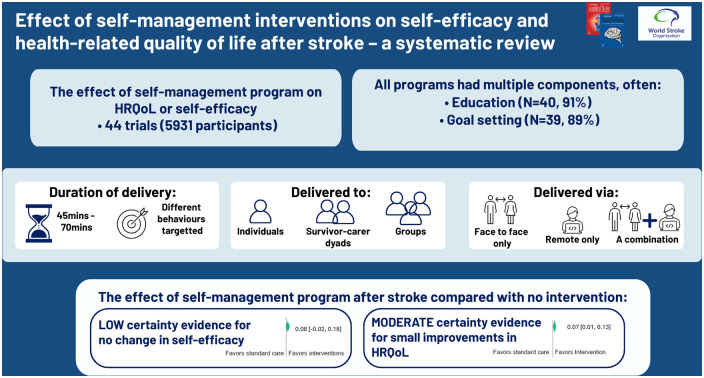

## Introduction

Disability is common after stroke and leads to reduced health-related quality of life (HRQoL).^1^ Post-stroke disability can be long-standing, so stroke requires long-term management. Accordingly, self-management interventions are recommended in many international stroke clinical guidelines, including those from Australia/New Zealand,^2^ Canada^3^ and United Kingdom/Ireland.^4^

Self-management interventions are designed to support people to make informed decisions^5^ and effectively manage health and recovery challenges,^6^ which contrasts with rehabilitation interventions which are typically designed to increase independence and reduce disability.^4^ Self-management interventions tend to be multi-component, incorporating strategies such as information provision, problem-solving, goal-setting, decision-making, self-monitoring, coping and activities to maintain or improve function.^7,8^ Accordingly, self-management interventions may improve health-related quality of life (HRQoL) through increased autonomy, enhanced coping strategies, and improved symptom management. Peer support networks have also been identified as important.^9^

Self-efficacy is a key construct underpinning many self-management interventions.^10^ Self-efficacy is an individual’s belief in their capability to accomplish specific tasks or overcome challenges,^11^ and is considered the major mechanism by which self-management skills are transformed into self-management actions.^5^ Following stroke, self-efficacy is essential for survivors’ active engagement in problem solving, making informed decisions and taking control of their health.^6,12^ Further, higher self-efficacy after stroke is associated with a range of outcomes, including less depression, more functional independence and lower risk of falling,^6^ so can be viewed as an important outcome as well as a mediator of self-management behaviors.

Given the increasing awareness of the importance of self-management after stroke, as demonstrated by inclusion in international clinical guidelines and an increasing volume of research trials, it is important to have up-to-date knowledge (a Cochrane review was published on this topic in 2016).^7^ The aim of this review is to update the evidence regarding the effectiveness of self-management interventions for improving self-efficacy and HRQoL in survivors of stroke.

## Methods

### Search strategy

The protocol was registered with the International Prospective Register of Systematic Reviews (CRD42023440168). We searched databases Medline, Emcare, CINAHL, Web of Science, Scopus, Cochrane Library, and trial registries from inception to 13/12/2024. (see Supplementary Table 1)

### Study selection

Randomized controlled trials (RCTs), comparing the effect of a self-management intervention to no intervention (or other intervention) for survivors of stroke on self-efficacy or HRQoL were included. We used the definition of self-management interventions reported in the 2016 Cochrane review by Fryer,^7^ wherein at least one of the following components was included: problem-solving, goal-setting, decision-making, self-monitoring, coping with the condition, or an alternative method designed to facilitate behavior change and improvements in physical and psychological functioning. Exclusion criteria: trials where <75% of participants were survivors of stroke and authors were unable to provide data for survivors of stroke only; self-management intervention was provided in conjunction with another intervention and the effects of interventions could not be separated; only carers or health providers recruited.

Titles and abstracts were exported to Covidence (https://www.covidence.org/), where they were screened independently by two reviewers. Full-text articles were reviewed independently by two reviewers. Disagreements were resolved through discussions.

### Data extraction

Two authors independently extracted data including descriptions of self-management interventions including components of self-management (problem-solving, goal setting, decision-making, self-monitoring, social networks, and coping strategies), theoretical rationale, mode of delivery, duration and frequency, and description of the comparator intervention. In RCTs with more than two arms, we classified the routine care arm as control and the most intensive self-management intervention arm as the intervention.

We extracted self-efficacy and HRQoL outcomes at baseline, 3-, 6-, and 12-month timepoints. Where multiple measures for one outcome were presented within a study, we selected EuroQol (EQ5-D) utility score as the HRQoL outcome measure and Stroke Self-Efficacy Questionnaire (SSEQ) as the self-efficacy measure.

We extracted outcome data as means and standard deviations for each time point. When necessary, we calculated means and standard deviations from reported data by multiplying the standard error with the square root of the number of participants, or by using mean, 95% confidence interval (CI) and sample sizes. We contacted trial authors if data were not published on relevant time points (baseline and 3 months), or when we were unable to calculate means and standard deviations for key outcomes.

### Assessment of risk of bias

Two reviewers independently assessed the risk of bias using the Cochrane Collaboration criteria.^13^ We evaluated heterogeneity by observing forest plots, calculating the I^2^ statistic and using Cochrane Q-test, with I^2^ > 75% deemed considerable heterogeneity.^14^ We tested publication bias and small study effects by using regression based Egger’s test and funnel plots.

### Measures of treatment effects

Data analyses were conducted using Stata (SE Version 18.0) for each outcome. Meta-analyses were performed to compare the estimates from baseline to each time point (3, 6, 12 months) using random effect model to consider random variability within studies and calculate differences between studies.^15^ Because different outcome measures were used for self-efficacy and HRQOL across trials, we estimated standardized mean differences (SMD) between groups using Cohens’ d formula. The results of the meta-analyses are presented as forest plots with effect sizes as SMDs and their 95% CIs. Sensitivity analyses were conducted by removing studies with high risk of bias from more than one source to check inconsistencies in the estimates.

We used meta-regressions to explore associations between effectiveness of the intervention and delivery method (face-to-face only versus element of telehealth); format (individual only versus element of groupwork); and duration of intervention (⩽7 h versus >7 h).

### Assessing certainty of evidence

Two authors independently assessed the certainty of the evidence for each outcome at each time point using the five GRADE considerations (study limitations, consistency, imprecision, indirectness, and publication bias).^16^

## Results

### Study selection and characteristics

**Figure 1.**
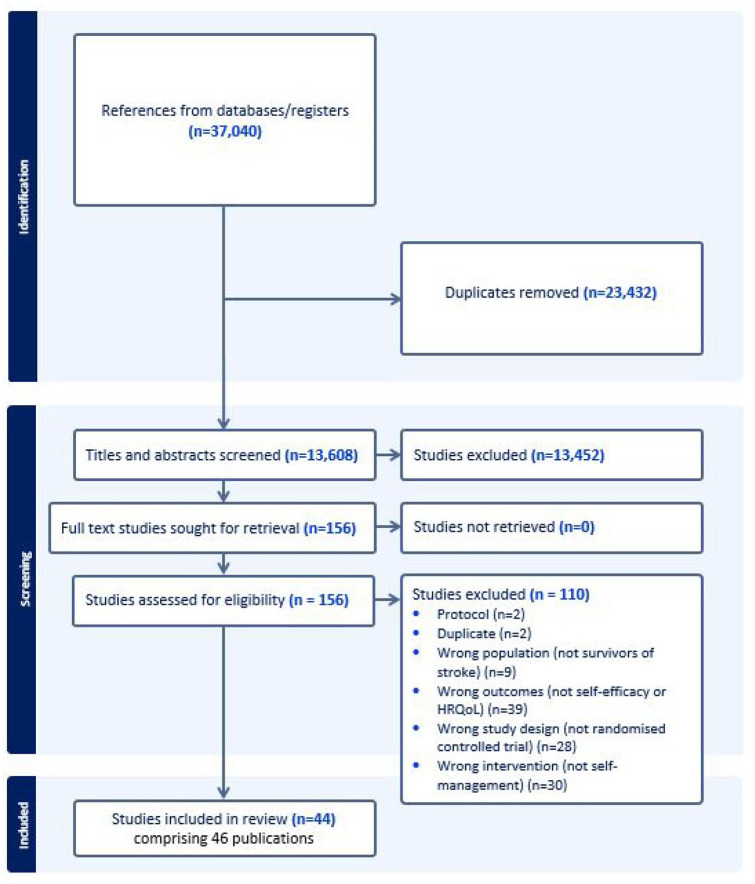
PRISMA flow diagram of search process.

After removing duplicates, 13,608 titles and abstracts were screened and 156 full text articles were reviewed. Two cluster-RCTs^17,18^ and 38 RCTs (44 trials, 46 publications)^19–63^ published in 15 countries (most commonly the United Kingdom, Australia and United States) between 2007 and 2024 were included (See Figure 1). There were 5931 participants (36% female) across all included trials, with a mean age of 64 years. Time since stroke varied from 14 days to 3 years, See Table 1. Most studies were funded by government agencies, healthcare or academic institutions and reported no conflicts of interest (Supplementary Table 2).

**Table 1.** Participant characteristics.

Author Year (Country)	Population, n (%)			Age, mean (SD)		Gender, female, n (%)		Time since stroke	Outcome measure (scale)	Timepoints
	Total	I	C	I	C	I	C			
Aben 2013 (Netherlands)	153	77	76	57.9 (9)	58.3 (10.4)	33 (42.8)	36 (47.4)	53.9 (37.2) months	HRQoL (EQ5D; WHOQoL)	Baseline ⩽10 days post-intervention 6, 12 months
Adamit 2023 (Israel)	49	33	33	64.6 (8.2)	64.4 (10.8)	11 (33.3)	15 (45.5)	>3 years	Self-efficacy (NGSE)	Baseline 10 weeks 3 months
Amiri 2022 (Iran)	72	36	36	69.1 (8.6)	67.7 (8.3)	14 (38.9)	19 (52.8)	NR	Self-efficacy (SSES)	Post-intervention 3 months
Barker-Collo 2015 (New Zealand)	386	193	193	NR	NR	NR	NR	⩽28 days	HRQoL(SF-36)	28 days 3, 6, 12 months
Bragstad 2020 (Norway)	322	166	156	66.8 (12.1)	65.7 (13.3)	67 (40.4)	65 (41.7)	4-8 weeks	HRQoL(SAQoL-39 g)	4-6 weeks 6, 12 months
Brauer 2022 (Australia)	119	52	49	62 (11)	64 (9)	12 (20)	13 (22)	I: 28(15) C: 27(16)	HRQoL (EQ-5D) Self-efficacy (Ambulatory self-confidence questionnaire)	Baseline 2 months, 6 months
Brouwer-Goossensen 2022 (Netherlands)	136	68	68	64 (13)	62 (14)	22 (32.3)	29 (42.6)	NR	Self-efficacy (SES)	Baseline 6 months
Cadilhac 2011 (Australia)	143	47^a^ 48	48	68 (12)	69 (11)^a^ 71 (12)	27 (56)	29 (60)^a^ 29 (62)	12 months	HRQoL(AQoL)	Baseline 6 months
Cadilhac 2020 (Australia)	54	25	29	69 (11)	68 (10)	10 (40)	11 (38)	2-24 months	HRQoL(EQ-5D-3 L)	Baseline 1 month
Chen 2019 (China)	144	72	72	65.9 (12.8)	64.8 (9.9)	20 (27.8)	18 (25)	3 months after discharge	Self-efficacy (SSEQ)	Baseline Discharge 1, 3 months
Damush 2011 (USA)	63	30	33	67.3 (12.4)	64 (8.4)	-	1 (3) ^b^	⩽1 month	HRQoL(SSQoL)	Baseline 3, 6 months
Damush 2016 (USA)	174	86	88	62.1 (9.4)	60.4 (9.5)	3 (3.4)	2 (2.3)	⩽12 months	HRQoL(SSQoL) Self-efficacy (GSES)	Baseline 6 months
Devasahayam 2024 (Canada)	57	29	28	61.7 (13.5)	59.9 (13.6)	12 (41)	14 (50)	I:106 (66) days, C:129 (83) days	Self-efficacy (Short Self-Efficacy for Exercise Scale)	Baseline
Fu 2020 (New Zealand)	400	270	130	71.4 (12.6)^a^ 71.7 (12.6)	73 (12.2)	58 (44)^a^ 53 (38.4)	55 (42.3)	45.5 (25.8) days	HRQoL(SF-36, SF-12, EQ5D)	Baseline 6 months
Harel-Katz 2020 (Israel)	60	31	29	65.5 (52.7)	66 (45.9)	5 (25)	4 (21)	I:113.1 (68.3) days C:147.6 (156.7) days	Self-efficacy (SEMCD)	Baseline 6, 12 months
Harwood 2012 (New Zealand)	172	94	78	61.4 (13.6)	61.3 (14.8)	49 (52.1)	41 (52.5)	6-12 weeks	HRQoL(SF-36)	Baseline 6 months
Heron 2019 (UK)	40	14^a^ 14	12	65.7 (13)^a^ 63.3 (9.6)	69.7 (14.7)	16 (40)^b^	-	⩽4 weeks	HRQoL(EQ-5D-5 L)	Baseline 3 months
Hoffmann 2015 (Australia)	36	11^a^ 12	10	63.6 (13)^a^ 60.8 (11.7)	57 (14.2)	4 (36.4)^a^ 3 (25)	4 (14)	NR	HRQoL (SAQoL-39 g) Self-efficacy (SSES)	Baseline I: 1 week, 3 months C: 2 months 5 months.
Jones 2016 (UK)	78	40	38	61.8 (16)	68.8 (10.3)	20 (50)	13 (34.2)	I:76(45-131 days), C: 116(46-171 days)	HRQoL(SAQoL-39 g; SF-12) Self-efficacy (SSES)	Baseline 6 weeks, 3 months
Kalav 2021 (Turkey)	68	34	34	55.9 (11.4)	58.9 (13.8)	12 (35.3)	12 (35.3)	NR	HRQoL(SSQoL) Self-Efficacy (SSES)	Baseline 3 months
Kendall 2007 (Australia)	100	42	58	66 (10.7)^c^	^-^	33 (33)^b^	-	NR	HRQoL(SSQoL) Self-efficacy (SES)	Baseline 3, 6, 9, 12 months
Kessler 2017 (Canada)	21	10	11	71 (13.2)	64.9 (16.3)	5 (50)	5 (45.4)	C:60.6(87.6) weeks I:29.2(18.2) weeks	Self-efficacy (GSAB–DFI)	Baseline Post-intervention 6 months
Lee 2023 (USA)	15	8	7	55 (44-76)^d^	53.5 (46-71)^d^	1 (12.5)^b^	-	I:4.6 (4.6) days C:4.3(1.9) days	Self-efficacy (PS-SES)	Baseline 3 months
Li 2024 (USA)	24	11	13	62 (11)	57 (12)	5 (45)	5 (38)	1245(1079) days	Self-efficacy (PS-SES and Patient-Reported Outcomes Measurement Information System’s Self-Efficacy)	Baseline 3 months
Lin 2022 (China)	70	35	35	60.8 (11)	62.5 (10.9)	16 (22.9)	18 (25.7)	NR	HRQoL(SSQoL-12) Self-efficacy (SSES)	Baseline 3, 6 months
Lo 2018 (Hong Kong)	128	64	64	67.5 (11.9)^c^	-	53 (41)^b^	-	45(26.2) days	HRQoL(SSQoL) Self-efficacy (SSES)	Baseline 2 months
Lo 2023a (Hong Kong)	335	169	166	62.5 (9.9)	63.1 (9.8)	64 (38.6)	66 (39.1)	C: 1.4 months (0.3–7.0) I: 1.33 months (0.3–7.5)	Self-efficacy (SSEQ)	Baseline 3, 6 months
Lo 2023b (Hong Kong)	134	67	67	63.4 (14.1)	64.8 (11.2)	27 (40.3)	29 (43.3)	4.2(5.1) years	Self-efficacy (SSEQ) HRQoL(SSQoL)	Baseline 8 weeks
Lund 2011 (Norway)	99	48	51	75 (7.2)	79 (6.5)	17 (43.6)	27 (57.4)	I: 161(178) days C; 137(124) days	HRQoL(SF-36)	Baseline 9 months
Mayo 2015 (Canada)	186	93	93	61 (12)	65 (11)	36 (38.7)	37 (39.8)	I:2.5(2.2) years C:3.1(3.1) years	HRQoL(EQ-5D)	Baseline 3 months
McKenna 2015 (UK)	25	12	13	62.2 (13.6)	67.4 (10.6)	4 (36.4)	7 (53.8)	NR	HRQoL(EQ-5D) Self-efficacy (SES; SSES)	Baseline 6 weeks 3 months
Minshall 2020 (Australia)	89	50	39	67 (13.7)	69 (11.9)	22 (52)	11 (35)	NR	HRQoL(AQoL, EQ-5D) Self-efficacy (GSES)	Baseline 3, 6, 12 months
Palleson 2024 (Denmark)	69	36	33	75 (6)	76 (6)	19 (56)	14 (44)	I:68(90) days, C:60(34) days	Self-efficacy (SSEQ) HRQoL(SSQoL-12)	Baseline 3, 9 months
Sabariego 2013 (Germany)	260	130	130	55.3 (12.6)	59.3 (12.7)	41 (37.3)	57 (44.3)	150.4(519.7) days	Self-efficacy (LSES) HRQoL(WHO-QoL, EQ5D-VAS)	Baseline, post-intervention, 6 months
Sahely 2024 (UK)	24	12	12	63 (16.53)	71 (13.04)	6 (50)	4 (33)	4.3(1.57) months	Self-efficacy (SSEQ)	Baseline 3, 6 months
Sakakibara 2022 (Canada)	126	64	62	69.1 (10.2)	62.7 (9.2)	26 (40.6)	13 (21)	217(108) days	HRQoL(SF-36)	Baseline 6, 12 months
Shaw 2020 (UK)	573	285	288	71 (60-77)^d^	71 (62-79)^d^	111 (39)	120 (41.7)	C:71.70(10.8) days I:77.5(12.7) days	HRQoL(EQ5D)	Baseline 12, 24 months
Sit 2018 (Hong Kong)	210	105	105	67.8 (14.2)	70.7 (16.9)	50 (47.6)	50 (47.6)	NR	Self-efficacy (SSES, Self-efficacy in illness management)	Baseline 1 week, 6 weeks 3 Months
Tielemans 2015 (Netherlands)	113	55	58	55.2 (8.9)	58.8 (8.7)	22 (40)	32 (55.2)	18.8(28.4) months	HRQoL (SSQoL) Self-efficacy (GSES)	Baseline 3, 9 months
Towfighi 2020 (USA)	100	49	51	60 (7)	57 (10)	20 (40.8)	18 (35.3)	⩽3 months	HRQoL(SF-6D)	Baseline 6 months
Tsai 2024 (Taiwan)	82	40	42	59.3 (14.1)	61.8 (14.5)	20 (50)	13 (31)	I:3.9 (1.8) months, C:3.6 (1.8) months	Self-efficacy (SEMCD) HRQoL(SF-12)	Baseline 1 month
Visser 2014 Netherlands and (Belgium)	166	88	78	52.2 (9.7)	54.1 (10.7)	33 (37.5)	45 (57.7)	7.3(1.1) months	HRQoL(SSQoL; EQ-5D-5 L)	Baseline 10 days post-intervention 6, 12 months
Wolf. 2016 (USA)	185	99	86	57 (10)	59 (7.7)	35 (53)	16 (51.6)	I:54(66.4), C:50(58) months	Self-efficacy (CDSES) HRQoL(WHOQoL-bref)	
Wolf 2017 (USA)	71	35	36	57.7 (11.6)	60.6 (10.6)	9 (25)	14 (38.9)	17.1(15.2)	Self-efficacy (CDSES) HRQoL(WHOQoL-bref)	Baseline Post intervention 6 months

a Two intervention streams.

b Total female population (groups not reported).

c Total age population (groups not reported).

d Interquartile range reported.

AQoL: Assessment of Quality of Life; C: Control; CDSES: Chronic Disease Self-Efficacy Scale; EQ-5D-3 L: EuroQol-5 dimension-3 level; EQ-5D: EuroQol-5 dimension; GSES: General self-efficacy scale; GSAB–DFI: Goals Systems Assessment Battery–Directive Functions Indicators I: Intervention; LSES: Liverpool Self-Efficacy Scale; NGSE: New General Self-Efficacy Scale; NR: Not reported; PS-SES: Participation strategies self-efficacy scale; SAQoL-39 g: Stroke and Aphasia Quality of Life scale-39 g; SEMCD: Self-efficacy for Managing Chronic Disease scale; SES: Self-efficacy Scale; SF-12: 12-Item Short-Form Health Survey; SF-36: 36-Item Short-Form Health Survey; SF-6D: Short form-6D; SSES: Stroke self-efficacy scale; SSQoL: Stroke-Specific Quality of Life Scale; UK: United Kingdom; USA: United States of America; VAS: Visual Analog Scale.

In all trials, self-management interventions contained multiple components. Education (N = 40, 91%) and goal setting (N = 39, 89%) were the most common components, and over half of the trials included the components of self-monitoring (N = 30, 68%) and problem-solving (N = 26, 59%). See Supplementary Table 3 for intervention components. Social cognitive theory^64^ (N = 9, 20%) or the principles of self-efficacy, a construct within social cognitive theory^64^ (N = 8, 18%) was the most cited theoretical rationale for the included interventions. Nearly half (N = 20, 45%) of the trials did not present a theoretical foundation for the intervention.

Self-management interventions were delivered to individual survivors of stroke (N = 18, 41%), groups of survivors (N = 15, 34%), combination of individual and group delivery to survivors (N = 5, 11%) and individually to survivor-carer dyads (N = 6, 14%). Interventions were delivered entirely face-to-face (N = 28, 64%), entirely by phone or video-conferencing software (N = 7, 16%) or using a combination of face-to-face and virtual delivery (N = 9, 20%). Resources such as workbooks, videos/DVDs and self-monitoring tools such as diaries, pedometers or blood pressure monitors were provided in 16 (36%) trials. Health professionals (most often nurses or allied health professionals) delivered interventions in all trials except three, which used trained research assistants^23,33^ and recreation and exercise therapists.^47^ Total intervention time ranged from 45 min to 72 h (median 7.5 h), and intervention durations ranged from a single session to 18 months (median 8 weeks) (Supplementary Table 4).

Self-management was compared to attention control (education,^19,29,30,50,54^ memory training^51^ or cardiorespiratory training)^18^ in seven trials, to written education or a single education session in three trials^31,33,60^ and to usual or routine care in the remaining 34 trials. Routine care was variable and poorly defined.

### Risk of bias

All trials had a high risk of bias due to not blinding participants or personnel, which is not possible in the design of these trials (Supplementary Table 5). One trial had high risk of bias as outcome data were collected by non-blinded assessors.^49^ Ten trials^21,27,32,34,37,41,48,59,60,62^ were deemed to have “other” source of bias due to small sample size (<62 participants, based on sample size power calculation using SSEQ).^38^

### Outcome measures

Twenty-seven trials included a measure of self-efficacy, 30 trials included a measure of HRQoL, and 14 trials included both outcomes (Table 1).

### Effectiveness of self-management interventions

#### Self-efficacy

**Figure 2.**
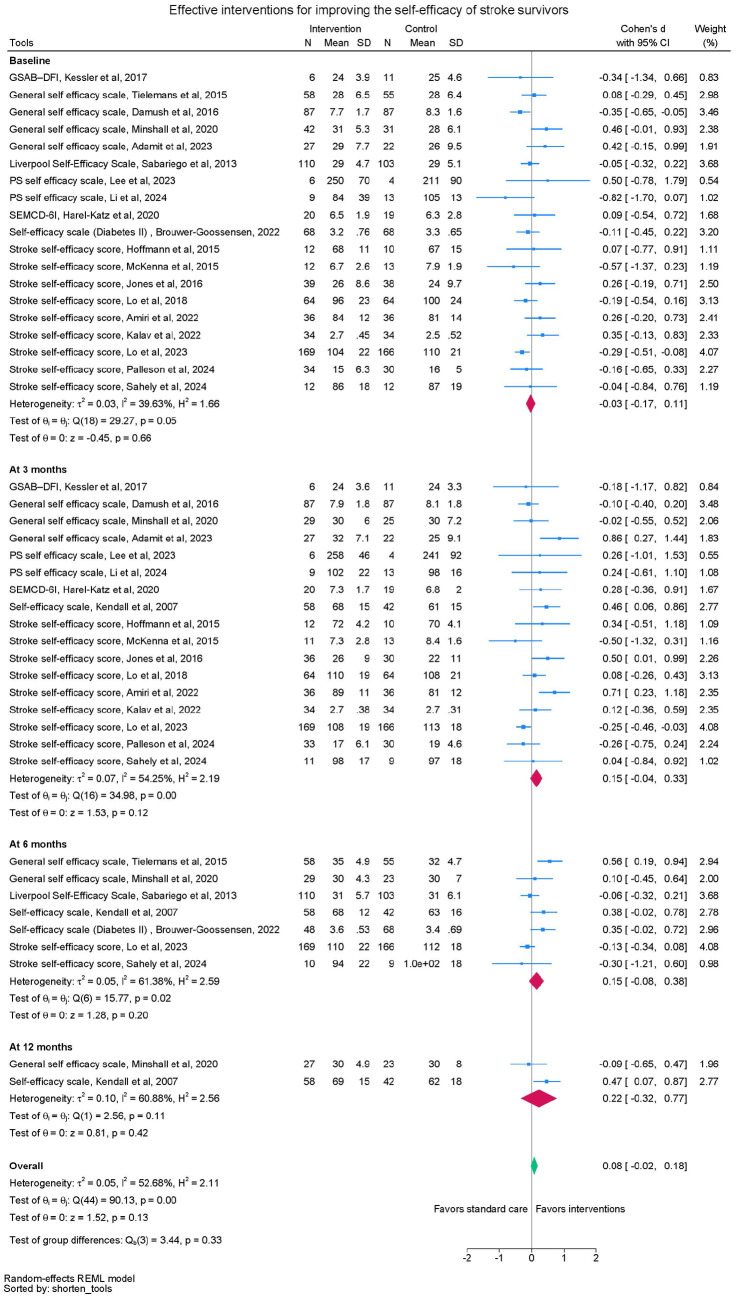
Self-efficacy meta-analysis.

Twenty trials were included in the meta-analyses to investigate the effect of self-management interventions on self-efficacy (Figure 2). Groups were similar at baseline. There were no significant differences between groups in self-efficacy scores at 3 months (SMD = 0.15, 95%CI: -0.04 to 0.33, 17 trials, n = 1263), 6 months (SMD = 0.15, 95%CI: -0.08 to 0.38, seven trials, n = 948), 12 months (SMD = 0.22, 95%CI -0.32 to 0.77, two trials, n = 150) or when data from all timepoints were pooled (SMD 0.08, 95%CI -0.02 to 0.18) (See Table 2). Moderate heterogeneity (I^2^ between 59-65% at the different time points) was observed, indicating variability in effect-sizes estimates.

**Table 2.** Summary of findings.

Self-management interventions compared with no intervention or another intervention for survivors of stroke			
**Population**: survivors of stroke **Setting:** hospital or community **Intervention:** self-management intervention^a^ **Comparison:** no intervention, “usual” care, or another intervention			
Outcomes	Relative effect: Standard mean difference (95% CI)	Number of participants (studies)	Certainty of the evidence (GRADE)
**Self-efficacy at 3** **months**	0.15 (–0.04 to 0.33)	1263 (17 trials)	⊕⊕⊝⊝Low^b,c^
**Self-efficacy (pooled)**	0.08 (–0.02 to 0.18)	1761 (20 trials)	⊕⊕⊝⊝Low^c,d^
**HRQoL at 3** **months**	0.10 (–0.00 to 0.20)	1613 (14 trials)	⊕⊕⊕⊝Moderate^c^

a. Self-management interventions varied, but contained at least one of the following: problem-solving, goal-setting, decision-making, self-monitoring, coping or an alternative method designed to facilitate behavior change.

b. Serious unexplained inconsistency (moderate heterogeneity I^2^ = 59%).

c. Serious imprecision (95% CI cross the decision-making threshold).

d. Serious unexplained inconsistency (moderate heterogeneity I^2^ = 53%).

e. Serious unexplained inconsistency (moderate heterogeneity I^2^ = 42%).

Regression-based test of small study effects (Egger’s test: z = 0.57, p = 0.57) and symmetrical shape of Funnel plots (Supplementary Figure 1) indicated no significant effect of publication bias in the analyses. The results from sensitivity analyses where data from trials with a high risk of bias from more than one source were removed, showed similar results at all time points as the initial analyses (Supplementary Figure 2) except for self-efficacy at 12 months, which only included data from one trial, and indicated a significant benefit for the treatment group (SMD = 0.47, 95%CI 0.07 to 0.87, one trial, n = 100).

#### Quality of life

**Figure 3.**
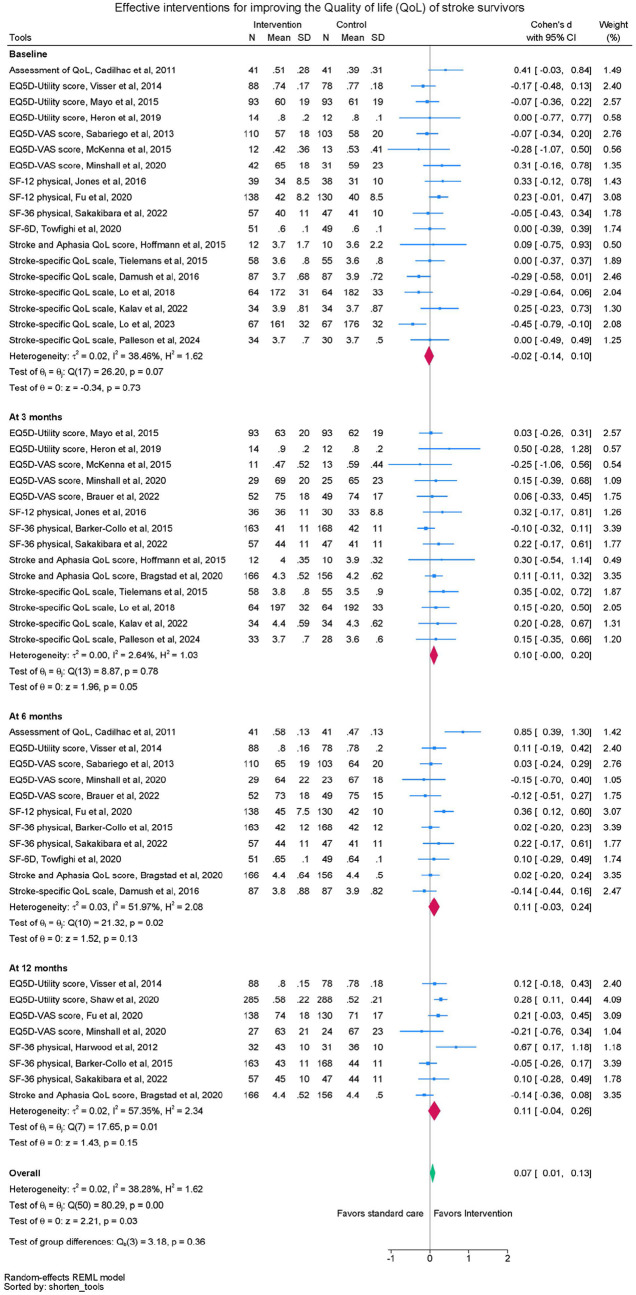
Quality of life meta-analysis.

Twenty-two trials were included in the meta-analyses comparing the effect of self-management intervention compared to control on HRQoL (Figure 3). Intervention and control groups were similar at baseline. No significant differences in standardized HRQoL scores were observed between groups at 3 months (SMD 0.10, 95% CI -0.00 to 0.20, p = 0.05, 14 trials, n = 1613), 6 months (SMD 0.11, 95% CI -.0.03 to 0.24, 11 trials, n = 1913) or 12 months (SMD 0.11, 95% CI -0.04 to 0.26, eight trials, n = 1878), but there was a small effect when data across timepoints were pooled (SMD 0.07, 95% CI 0.01 to 0.13) (see Table 2). Heterogeneity was not apparent at 3 months (I^2^ = 4.16%) but medium heterogeneity was observed at 6 and 12 months (I^2^ = 58% and 57% respectively).

The symmetrical funnel plot Supplementary (Figure 3) and test of small study effects (Egger’s test: z = 0.99, p = 0.32) demonstrated no signs of publication bias in HRQoL analyses. The sensitivity analyses which only included data from trials with high risk of bias from one source only (non-blinding of participants and personnel) similarly indicated no significant difference between groups at the three timepoints, and a marginal benefit in favor of the intervention group in the pooled analysis (Supplementary Figure 4).

### Meta-regression

#### Self-efficacy

Inclusion of virtual (telephone or teleconference) intervention delivery was associated with significantly reduced intervention effectiveness on self-efficacy compared to exclusively face-to-face delivery (reduction of 0.33 points in scores, 95%CI -0.56 to -0.11), and interventions with total duration longer than 7 h were more effective than shorter interventions (slope 0.34, 95%CI 0.10 to 0.57). Delivery to individuals versus groups was not significantly associated with self-efficacy scores. (Supplementary Table 6).

#### HRQoL

Inclusion of virtual delivery (versus entirely face-to-face), delivery to individuals versus groups and duration of intervention (⩽7 h versus >7 h) were not significantly associated with HRQOL scores (Supplementary Table 6).

### Certainty of evidence

The certainty of evidence when applying the GRADE criteria for the effect of self-management interventions compared to control on self-efficacy was low, due to inconsistency between trial results and imprecision. The certainty of evidence for the effect of self-management interventions compared to control on HRQoL was moderate (downgraded at 3 months due to imprecision, downgraded on pooled results due to inconsistency).

## Discussion

In this systematic review with meta-analyses, we found low certainty evidence that self-management interventions after stroke compared to usual care or another intervention did not significantly improve self-efficacy, but pooled effect sizes demonstrated moderate certainty evidence of a marginal significant effect on HRQoL. The evidence for HRQoL was complex to summarize and interpret since it included transformed summary data and raw scores from the trials. We also found that face-to-face interventions, and interventions that took more than 7 h to deliver, were associated with better self-efficacy, but not HRQoL outcomes.

Our findings contrast somewhat to those of a Cochrane systematic review published in 2016,^7^ which reported low certainty evidence that self-management programs improved self-efficacy and HRQoL. This review, published 9 years ago, included 14 trials with 1863 participants. We were able to upgrade the certainty of evidence for HRQoL due to the proliferation of stroke self-management research; we included a further 36 trials with an additional 4941 participants. Differences may also have arisen due to the different analytical approaches. We analyzed outcome data according to pre-specified time points (3, 6, and 12 months) and combined scores from different self-efficacy and HRQoL outcome measures by standardizing the scores using standardized mean differences so we could report on each concept (self-efficacy, HRQoL) at each time point. In contrast, Fryer and colleagues^7^ reported analyses for individual outcome measures with the time point not specified.

The included self-management interventions had different theoretical underpinnings, targeted different behaviors, comprised different strategies and delivered different doses. Despite the interventions in over a third of the included trials reportedly being based on the principles of self-efficacy or Social Cognitive Theory (of which self-efficacy is a key construct),^64^ very few included trials reported delivering strategies known to improve self-efficacy, such as reflecting on success (mastery) or learning from other survivors (vicarious modeling).^6^ Very few described how the program was designed or the program logic (why the strategies within the program were selected and the anticipated mechanism of action). Instead, there was an apparent over-reliance on education strategies (included in 91% of trials), which on its own, tends not to be effective at changing self-management behaviors or attitudes after stroke.^65^ Further, when programs are designed without input from survivors and family members, the potential power of vicarious modeling is absent. Developers of future self-management interventions should build on the current body of evidence, and consider how to incorporate elements that promote self-efficacy (advice from survivors and family members) and support behavior change to promote self-management.

The role of self-management after stroke is becoming increasingly recognized, as demonstrated by inclusion in stroke rehabilitation guidelines from Australia/New Zealand, Canada, and United Kingdom/Ireland. Therefore, having clear evidence regarding which strategies are most effective to optimize self-efficacy and self-management skills is important. Our results indicate a benefit on self-efficacy by including group work and face-to-face delivery in self-management programs. Given the growing popularity and availability of online and virtual resources and programs, developers, deliverers and funders of online or virtual programs should consider incorporating and evaluating features such as emerging immersive virtual and augmented reality options that could replicate the benefits of face-to-face delivery. Other features, such as video stories by survivors about self-managing their health and wellbeing after stroke, have also been perceived as a way to enhance viewers’ self-efficacy.^44^

The strengths of this review are that robust methodology was followed, and we have synthesized data from 44 trials. Limitations include heterogeneity of the interventions delivered, outcome measures used, the targeted behaviors, time since stroke, and how and when outcomes were measured. Further, we did not collect data on stroke-related impairments or activity limitations of participants pre- or post-interventions.

## Conclusion

Self-management interventions for survivors of stroke do not consistently improve self-efficacy or HRQoL. Theoretically developed interventions and consensus on outcome measures to use would improve our ability to investigate effectiveness in future.

## Supplemental Material

sj-docx-1-wso-10.1177_17474930251340286 – Supplemental material for Do self-management interventions improve self-efficacy and health-related quality of life after stroke? A systematic review Supplemental material, sj-docx-1-wso-10.1177_17474930251340286 for Do self-management interventions improve self-efficacy and health-related quality of life after stroke? A systematic review by Elizabeth A Lynch, Katie Nesbitt, Aarti Gulyani, Raymond J Chan, Niranjan Bidargaddi, Dominique A Cadilhac, Billie Bonevski, Fiona Jones, Liam P Allan, Erin Godecke, Rebecca Barnden, Emily Brogan, Thoshenthri Kandasamy, Stacy Larcombe, Lemma N Bulto and Coralie English in International Journal of Stroke
