# Supplementary material for: Do self-management interventions improve self-efficacy and health-related quality of life after stroke? A systematic review
Source: Int J Stroke. 2025 Apr 24;20(7):786–800. doi: 10.1177/17474930251340286 (PMC12264303; doi:10.1177/17474930251340286)
Supplement: sj-docx-1-wso-10.1177_17474930251340286 – Supplemental material for Do self-management interventions improve self-efficacy and health-related quality of life after stroke? A systematic review [file sj-docx-1-wso-10.1177_17474930251340286.docx]

**Supplementary table 1. Search strategy**

| **#** | **Search: Ovid MEDLINE** |  |  |
| --- | --- | --- | --- |
| 1 | Independent Living/ |  |  |
| 2 | Home Environment/ |  |  |
| 3 | ((living adj2 (independent* or autonomous* or "at home")) or ((remaining or residing or own) adj2 home)).tw,kf. |  |  |
| 4 | ((community adj2 (dwelling* or living)) or "home dwelling" or "home based" or ((home or community) adj2 based)).tw,kf. |  |  |
| 5 | ((out adj2 hospital) or ((post or after) adj2 discharge*)).tw,kf. |  |  |
| 6 | or/1-5 |  |  |
| 7 | stroke/ or brain infarction/ or hemorrhagic stroke/ or ischemic stroke/ |  |  |
| 8 | (stroke* or "cerebral vascular event*" or "Cerebral vascular disease" or "brain infarction").tw,kf. |  |  |
| 9 | ((brain* or cerebr* or cerebell* or intracran* or intracerebral) adj3 (isch?emi* or infarct* or thrombo* or emboli* or occlus*)).tw,kf. |  |  |
| 10 | ((brain* or cerebr* or cerebell* or intracerebral or intracranial or subarachnoid) adj5 (haemorrhage* or hemorrhage* or haematoma* or hematoma* or bleed*)).tw,kf. |  |  |
| 11 | or/7-10 |  |  |
| 12 | self care/ or self-management/ |  |  |
| 13 | self efficacy/ |  |  |
| 14 | Self Administration/ |  |  |
| 15 | Adaptation, Psychological/ |  |  |
| 16 | "patient acceptance of health care"/ or patient compliance/ or patient participation/ or patient satisfaction/ or patient preference/ |  |  |
| 17 | (self adj2 (care or management or efficacy or adminstrat*)).tw,kf. |  |  |
| 18 | (patient adj2 (Compliance or participation or satisfaction or preference*)).tw,kf. |  |  |
| 19 | (coping or cope*).tw,kf. |  |  |
| 20 | or/12-19 |  |  |
| 21 | exp clinical trial/ or randomized controlled trial/ |  |  |
| 22 | ((randomized controlled trial or controlled clinical trial).pt. or randomized.ab. or placebo.ab. or drug therapy.fs. or randomly.ab. or trial.ab. or groups.ab.) not (exp animals/ not humans.sh.) |  |  |
| 23 | ("double blind" or placebo or allocated).mp. [mp=title, book title, abstract, original title, name of substance word, subject heading word, floating sub-heading word, keyword heading word, organism supplementary concept word, protocol supplementary concept word, rare disease supplementary concept word, unique identifier, synonyms, population supplementary concept word, anatomy supplementary concept word] |  |  |
| 24 | 21 or 22 or 23 |  |  |
| 25 | 6 and 11 and 20 and 24 |  |  |
| **#** | **Search: Ovid Emcare** | | |
| 1 | independent living/ | | |
| 2 | home environment/ | | |
| 3 | ((living adj2 (independent* or autonomous* or "at home")) or ((remaining or residing or own) adj2 home)).tw,kw. | | |
| 4 | ((community adj2 (dwelling* or living)) or "home dwelling" or "home based" or ((home or community) adj2 based)).tw,kw. | | |
| 5 | ((out adj2 hospital) or ((post or after) adj2 discharge*)).tw,kw. | | |
| 6 | or/1-5 | | |
| 7 | cerebrovascular accident/ or ischemic stroke/ | | |
| 8 | brain infarction/ | | |
| 9 | brain hemorrhage/ | | |
| 10 | (stroke* or "cerebral vascular event*" or "Cerebral vascular disease" or "brain infarction").tw,kw. | | |
| 11 | ((brain* or cerebr* or cerebell* or intracran* or intracerebral) adj3 (isch?emi* or infarct* or thrombo* or emboli* or occlus*)).tw,kw. | | |
| 12 | ((brain* or cerebr* or cerebell* or intracerebral or intracranial or subarachnoid) adj5 (haemorrhage* or hemorrhage* or haematoma* or hematoma* or bleed*)).tw,kw. | | |
| 13 | or/7-12 | | |
| 14 | self care/ | | |
| 15 | self concept/ | | |
| 16 | psychological adjustment/ | | |
| 17 | patient attitude/ or patient compliance/ or patient participation/ or patient satisfaction/ | | |
| 18 | patient preference/ | | |
| 19 | (self adj2 (care or management or efficacy or administrat*)).tw,kw. | | |
| 20 | (patient adj2 (Compliance or participation or satisfaction or preference*)).tw,kw. | | |
| 21 | (coping or cope*).tw,kw. | | |
| 22 | or/14-21 | | |
| 23 | randomized controlled trial/ or controlled clinical trial/ | | |
| 24 | clinical trial/ | | |
| 25 | ((randomized controlled trial or controlled clinical trial).pt. or randomized.ab. or placebo.ab. or randomly.ab. or trial.ab. or groups.ab.) not (exp animals/ not humans.sh.) | | |
| 26 | ("double blind" or placebo or allocated).mp. [mp=title, abstract, heading word, drug trade name, original title, device manufacturer, drug manufacturer, device trade name, keyword heading word] | | |
| 27 | or/23-26 | | |
| 28 | 6 and 13 and 22 and 27 | | |
| 29 | 6 and 13 and 22 | | |
| **#** | **Search: APA PsycInfo** | |  |
| 1 | home environment/ | |  |
| 2 | ((living adj2 (independent* or autonomous* or "at home")) or ((remaining or residing or own) adj2 home)).ti,ab,id. | |  |
| 3 | ((community adj2 (dwelling* or living)) or "home dwelling" or "home based" or ((home or community) adj2 based)).ti,ab,id. | |  |
| 4 | ((out adj2 hospital) or ((post or after) adj2 discharge*)).ti,ab,id. | |  |
| 5 | or/1-4 | |  |
| 6 | cerebrovascular accidents/ or cerebral hemorrhage/ or cerebral ischemia/ or subarachnoid hemorrhage/ | |  |
| 7 | (stroke* or "cerebral vascular event*" or "Cerebral vascular disease" or "brain infarction").ti,ab,id. | |  |
| 8 | ((brain* or cerebr* or cerebell* or intracran* or intracerebral) adj3 (isch?emi* or infarct* or thrombo* or emboli* or occlus*)).ti,ab,id. | |  |
| 9 | ((brain* or cerebr* or cerebell* or intracerebral or intracranial or subarachnoid) adj5 (haemorrhage* or hemorrhage* or haematoma* or hematoma* or bleed*)).ti,ab,id. | |  |
| 10 | or/6-9 | |  |
| 11 | self-care/ or self-management/ | |  |
| 12 | self-efficacy/ | |  |
| 13 | coping behavior/ | |  |
| 14 | client participation/ or treatment compliance/ | |  |
| 15 | client satisfaction/ | |  |
| 16 | (self adj2 (care or management or efficacy or adminstrat*)).ti,ab,id. | |  |
| 17 | (patient adj2 (Compliance or participation or satisfaction or preference*)).ti,ab,id. | |  |
| 18 | (coping or cope*).ti,ab,id. | |  |
| 19 | or/11-18 | |  |
| 20 | randomized controlled trials/ or exp clinical trials/ or randomized clinical trials/ or placebo/ | |  |
| 21 | ((randomized controlled trial or controlled clinical trial).pt. or randomized.ab. or placebo.ab. or randomly.ab. or trial.ab. or groups.ab.) not (exp animals/ not humans.sh.) | |  |
| 22 | ("double blind" or placebo or allocated).mp. [mp=title, abstract, heading word, table of contents, key concepts, original title, tests & measures, mesh word] | |  |
| 23 | 20 or 21 or 22 | |  |
| 24 | 5 and 10 and 19 and 23 | |  |
| 25 | 5 and 10 and 19 | |  |
| **#** | **Search: CINAHL** | |  |
| 1 | (MH "Community Living") | |  |
| 2 | (MH "Home Environment") | |  |
| 3 | TI ( ((living N1 (independent* or autonomous* or "at home")) or ((remaining or residing or own) N1 home)) ) OR AB ( ((livingN1 (independent* or autonomous* or "at home")) or ((remaining or residing or own) N1 home)) ) | |  |
| 4 | TI ( ((community N1(dwelling* or living)) or "home dwelling" or "home based" or ((home or community) N1 based)) ) OR AB ( ((community N1(dwelling* or living)) or "home dwelling" or "home based" or ((home or community) N1 based) ) | |  |
| 5 | TI ( ((out N1 hospital) or ((post or after) N1 discharge*)) ) OR AB ( ((out N1 hospital) or ((post or after) N1 discharge*)) ) | |  |
| 6 | S1 OR S2 OR S3 OR S4 OR S5 | |  |
| 7 | (MH "Intracranial Hemorrhage") OR (MH "Cerebral Hemorrhage") OR (MH "Stroke") OR (MH "Cerebral Infarction") OR (MH "Hemorrhagic Stroke") OR (MH "Ischemic Stroke") OR (MH "Embolic Stroke") | |  |
| 8 | TI ( (stroke* or "cerebral vascular event*" or "Cerebral vascular disease" or "brain infarction") ) OR AU ( (stroke* or "cerebral vascular event*" or "Cerebral vascular disease" or "brain infarction") ) | |  |
| 9 | TI ( ((brain* or cerebr* or cerebell* or intracran* or intracerebral) N2 (isch?emi* or infarct* or thrombo* or emboli* or occlus*)) ) OR AB ( ((brain* or cerebr* or cerebell* or intracran* or intracerebral) N2 (isch?emi* or infarct* or thrombo* or emboli* or occlus*)) ) | |  |
| 10 | TI ( ((brain* or cerebr* or cerebell* or intracerebral or intracranial or subarachnoid) N4 (haemorrhage* or hemorrhage* or haematoma* or hematoma* or bleed*) ) OR AB ( ((brain* or cerebr* or cerebell* or intracerebral or intracranial or subarachnoid) N4 (haemorrhage* or hemorrhage* or haematoma* or hematoma* or bleed*) ) | |  |
| 11 | S7 OR S8 OR S10 | |  |
| 12 | (MH "Self-Management") OR (MH "Self Care") | |  |
| 13 | (MH "Self-Efficacy") | |  |
| 14 | (MH "Self Administration") | |  |
| 15 | (MH "Adaptation, Psychological") | |  |
| 16 | (MH "Patient Compliance") OR (MH "Patient Satisfaction") | |  |
| 17 | (MH "Patient Preference") | |  |
| 18 | TI ( (self N1 (care or management or efficacy or adminstrat*)) ) OR AB ( (self N1 (care or management or efficacy or adminstrat*)) ) | |  |
| 19 | TI ( (patient N1 (Compliance or participation or satisfaction or preference*) ) OR AB ( (patient N1 (Compliance or participation or satisfaction or preference*) ) | |  |
| 20 | TI ( (coping or cope*) ) OR AB ( (coping or cope*) ) | |  |
| 21 | S12 OR S13 OR S14 OR S15 OR S16 OR S17 OR S18 OR S19 OR S20 | |  |
| 22 | (MH "Randomized Controlled Trials") | |  |
| 23 | TX allocat* random* OR (MH "Placebos") OR TX placebo* OR TX random* allocat* OR (MH "Random Assignment") OR TX randomi* control* trial* OR TX ( (doubl* n1 blind*) OR OR TX clinic* n1 trial* OR PT Clinical trial OR (MH "Clinical Trials+") | |  |
| 24 | S22 OR S23 | |  |
| 25 | S6 AND S11 AND S21 AND S24 | |  |
| 26 | S6 AND S11 AND S21 | |  |
| **#** | **Search: Scopus** | |  |
|  | Search string for RCTs  ( ( ( TITLE-ABS-KEY ( LIVING W/1 INDEPENDENT* OR AUTONOMOUS* OR "AT HOME" ) OR TITLE-ABS-KEY ( COMMUNITY W/1 DWELLING* OR LIVING ) OR TITLE-ABS-KEY ( HOME OR COMMUNITY W/1 BASED ) OR TITLE-ABS-KEY ( OUT W/1 HOSPITAL ) OR TITLE-ABS-KEY ( POST OR AFTER W/1 DISCHARGE* ) ) ) AND ( ( TITLE-ABS-KEY ( STROKE* OR "CEREBRAL VASCULAR EVENT*" OR "CEREBRAL VASCULAR DISEASE" OR "BRAIN INFARCTION" ) OR TITLE-ABS-KEY ( BRAIN* OR CEREBR* OR CEREBELL* OR INTRACRAN* OR INTRACEREBRAL W/2 ISCH?EMI* OR INFARCT* OR THROMBO* OR EMBOLI* OR OCCLUS* ) OR TITLE-ABS-KEY ( ( ( BRAIN* OR CEREBR* OR CEREBELL* OR INTRACEREBRAL OR INTRACRANIAL OR SUBARACHNOID ) W/4 ( HAEMORRHAGE* OR HEMORRHAGE* OR HAEMATOMA* OR HEMATOMA* OR BLEED* ) ) ) ) ) AND ( ( TITLE-ABS-KEY ( SELF W/1 CARE OR MANAGEMENT OR EFFICACY OR ADMINSTRAT* ) OR TITLE-ABS-KEY ( PATIENT W/1 COMPLIANCE OR PARTICIPATION OR SATISFACTION OR PREFERENCE* ) OR TITLE-ABS-KEY ( COPING OR COPE* ) ) ) ) AND ( TITLE-ABS-KEY ( RANDOM* OR TRIAL* OR PLACEBO OR GROUP* OR ASSIGNED OR ALLOCATED OR "DOUBLE BLIND*" ) )  Search string for no specific filter for study design  ( ( TITLE-ABS-KEY ( LIVING W/1 INDEPENDENT* OR AUTONOMOUS* OR "AT HOME" ) OR TITLE-ABS-KEY ( COMMUNITY W/1 DWELLING* OR LIVING ) OR TITLE-ABS-KEY ( HOME OR COMMUNITY W/1 BASED ) OR TITLE-ABS-KEY ( OUT W/1 HOSPITAL ) OR TITLE-ABS-KEY ( POST OR AFTER W/1 DISCHARGE* ) ) ) AND ( ( TITLE-ABS-KEY ( STROKE* OR "CEREBRAL VASCULAR EVENT*" OR "CEREBRAL VASCULAR DISEASE" OR "BRAIN INFARCTION" ) OR TITLE-ABS-KEY ( BRAIN* OR CEREBR* OR CEREBELL* OR INTRACRAN* OR INTRACEREBRAL W/2 ISCH?EMI* OR INFARCT* OR THROMBO* OR EMBOLI* OR OCCLUS* ) OR TITLE-ABS-KEY ( ( ( BRAIN* OR CEREBR* OR CEREBELL* OR INTRACEREBRAL OR INTRACRANIAL OR SUBARACHNOID ) W/4 ( HAEMORRHAGE* OR HEMORRHAGE* OR HAEMATOMA* OR HEMATOMA* OR BLEED* ) ) ) ) ) AND ( ( TITLE-ABS-KEY ( SELF W/1 CARE OR MANAGEMENT OR EFFICACY OR ADMINSTRAT* ) OR TITLE-ABS-KEY ( PATIENT W/1 COMPLIANCE OR PARTICIPATION OR SATISFACTION OR PREFERENCE* ) OR TITLE-ABS-KEY ( COPING OR COPE* ) ) ) | |  |
| **#** | **Search: Cochrane Library** | |  |
|  | ((living NEAR/1 (independent* OR autonomous* OR "at home")) OR ((remaining OR residing OR own) NEAR/1 home) OR ((community NEAR/1 (dwelling* OR living)) OR "home dwelling" OR "home based" OR ((home OR community) NEAR/1 based)) OR (out NEAR/1 hospital) OR ((post OR after) NEAR/1 discharge*))    (stroke* OR "cerebral vascular event*" OR "Cerebral vascular disease" OR "brain infarction" OR ((brain* OR cerebr* OR cerebell* OR intracran* OR intracerebral) NEAR/2 (isch?emi* OR infarct* OR thrombo* OR emboli* OR occlus*)) OR ((brain* OR cerebr* OR cerebell* OR intracerebral OR intracranial OR subarachnoid) NEAR/4 (haemorrhage* OR hemorrhage* OR haematoma* OR hematoma* OR bleed*)))    ((self NEAR/1 (care OR management OR efficacy OR adminstrat*)) OR (patient NEAR/1 (Compliance OR participation OR satisfaction OR preference*)) OR coping OR cope*) | |  |
| **#** | **Search: Web of Science** | |  |
|  | ((living NEAR/1 (independent* OR autonomous* OR "at home")) OR ((remaining OR residing OR own) NEAR/1 home) OR ((community NEAR/1 (dwelling* OR living)) OR "home dwelling" OR "home based" OR ((home OR community) NEAR/1 based)) OR (out NEAR/1 hospital) OR ((post OR after) NEAR/1 discharge*))    (stroke* OR "cerebral vascular event*" OR "Cerebral vascular disease" OR "brain infarction" OR ((brain* OR cerebr* OR cerebell* OR intracran* OR intracerebral) NEAR/2 (isch?emi* OR infarct* OR thrombo* OR emboli* OR occlus*)) OR ((brain* OR cerebr* OR cerebell* OR intracerebral OR intracranial OR subarachnoid) NEAR/4 (haemorrhage* OR hemorrhage* OR haematoma* OR hematoma* OR bleed*)))    ((self NEAR/1 (care OR management OR efficacy OR adminstrat*)) OR (patient NEAR/1 (Compliance OR participation OR satisfaction OR preference*)) OR coping OR cope*)  Random* OR placebo OR assigned OR group* OR trial* | |  |

**Supplementary Table 2. Funding sources of included trials**

| **Author** | **Funding source/s** |
| --- | --- |
| Aben 2013 | Kinder Fonds Adriaanstichting (Children’s fund Adriaanstichting) |
| Adamit 2023 | Kahn-Sagol-Maccabi Research and Innovation research grant, Steyer Family scholarship. |
| Amiri 2022 | Isfahan University of Medical Science. |
| Barker-Collo 2015 | New Zealand Health Research Council |
| Bragstad 2020 | European Union Seventh Framework Program; the South-Eastern Norway Regional Health Authority; and the Extra Foundation. The University of Oslo, Oslo University Hospital, the Inland Norway University of Applied Sciences, and UiT, the Arctic University of Norway, Narvik. |
| Brauer 2022 | National Health and Medical Research Council of Australia |
| Brouwer-Goossensen 2022 | Evidence Based Care for Nurses fund of the Erasmus University Medical Hospital |
| Cadilhac 2011 | J.O. and J.R. Wicking Trust, in-kind support from the National Stroke Foundation |
| Cadilhac 2020 | Monash University, Monash University Faculty Strategic Grant |
| Chen 2019 | National Natural Science Fund of China |
| Damush 2011 | Merit review grant from the VA Health Services Research and Development |
| Damush 2016 | VA HSRD Investigator Initiated Research Grant, HSRD VA Stroke QUERI Center |
| Devasahayam 2024 | Canadian Institutes of Health Research |
| Fu 2020 | Health Research Council of New Zealand |
| Harel-Katz 2020 | Scholarship from the “Foundation for promoting the Research of Aging” of University of Haifa and JDC Israel, excellence scholarship for PhD candidate from the Graduate Research Authority of University of Haifa, Israel. |
| Harwood 2012 | Health Research Council of New Zealand and the B Basham Medical Charitable Trust |
| Heron 2019 | The National Institute for Health Research and British Association of Sport and  Exercise Medicine |
| Hoffmann 2015 | The University of Queensland and Griffith University |
| Jones 2016 | The National Institute for Health Research (Research for Patient Benefit Programme) |
| Kalav 2022 | Akdeniz University Scientific Research Projects Coordination Unit |
| Kendall 2007 | Australian Research Council, the Motor Accident Insurance Commission of  Queensland, the Acquired Brain Injury Outreach Service and the Brisbane South Division of General Practice |
| Kessler 2017 | University of Ottawa Brain and Mind Research Institute |
| Lee 2023 | American Occupational Therapy Foundation Intervention Research Grant |
| Li 2024 | American Occupational Therapy Foundation, the National Center for Medical Rehabilitation Research, the National Institute of Mental Health and the Washington University Mobile Health Research Core, part of the Institute of Clinical and Translational Sciences, funded by the National Center for Advancing Translational Sciences. |
| Lin 2022 | Flinders University |
| Lo 2018 | No specific funding for the research |
| Lo 2023a | Health and Medical Research Fund, Food and Health Bureau, the Government of the Hong Kong Special Administrative Region |
| Lo 2023b | Health Care and Promotion Scheme, Food and Health Bureau, The Government of  the Hong Kong Special Administrative Region |
| Lund 2012 | The Eastern Health Region in Norway, the Department of Geriatric Medicine at Oslo University Hospital and the Norwegian Women’s Public Health Association. Oslo University College and the Norwegian Association for Occupational Therapists. |
| Mayo 2015 | No funding from public, commercial or not-for-profit sectors |
| McKenna 2015 | Northern Ireland Chest, Heart and Stroke |
| Minshall 2020 | Collaborative Research Network |
| Pallesen 2024 | The Velux Foundation |
| Sabariego 2013 | German Federal Ministry of Education and Research |
| Sahely 2024 | No specific funding for the research |
| Sakakibara 2022 | Canadian Institutes of Health Research Postdoctoral Fellowship and Operating Grant; Michael Smith Foundation for Health Research Postdoctoral Fellowship and Senior Scholar Award; Canada Research Chair Program; Pfizer/Heart and Stroke  Foundation Chair in Cardiovascular Prevention Research at St. Paul’s Hospital; Michael Smith Foundation for Health Research Health Professional Investigator Award; and the Heart and Stroke Foundation Canadian Partnership for Stroke Recovery Operating Grant. |
| Shaw 2020 | National Institute for Health Research |
| Sit 2018 | Health and Health Services Research Fund, Food and Health Bureau, Hong Kong SAR Government |
| Tielemans 2015 | Dutch VSBFonds and the Dutch Heart Foundation |
| Towfighi 2020 | American Heart Association National Scientist Development Award |
| Tsai 2024 | The National Science and Technology Council, Taiwan |
| Visser 2014 | National Initiative Brain and Cognition (NIBC; The Healthy Brain, Program Cognitive Rehabilitation), Stichting Coolsingel, and Erasmus MC Cost-Effectiveness Research |
| Wolf 2016 | National Institute on Disability and Rehabilitation Research within the Rehabilitation Research and Training Center on enhancing the functional and employment outcomes of individuals who experience a stroke. |
| Wolf 2017 | National Center for Medical Rehabilitation Research (NCMRR) in the National Institute of Child Health and Human Development (NICHD) of the National Institutes of Health (NIH) |

**Supplementary Table 3. Components of self-management interventions**

| Author |  | Components of self-management interventions | | | | | | |
| --- | --- | --- | --- | --- | --- | --- | --- | --- |
|  | Problem-solving | | Goalsetting | Decision-making | Self-monitoring | Education | Social networks | Coping strategies |
| Aben 2013 |  | | ✓ |  |  | ✓ |  |  |
| Adamit 2023 |  | | ✓ | ✓ | ✓ | ✓ |  |  |
| Amiri 2022 | ✓ | | ✓ | ✓ | ✓ | ✓ |  |  |
| Barker-Collo 2015 |  | | ✓ |  | ✓ | ✓ |  |  |
| Bragstad 2020 | ✓ | |  |  |  | ✓ | ✓ | ✓ |
| Brauer 2022 | ✓ | | ✓ |  | ✓ | ✓ |  | ✓ |
| Brouwer-Goossensen 2022 | ✓ | | ✓ | ✓ | ✓ | ✓ |  |  |
| Cadilhac 2011 | ✓ | |  |  | ✓ | ✓ | ✓ | ✓ |
| Cadilhac 2020 |  | | ✓ |  | ✓ | ✓ |  | ✓ |
| Chen 2019 | ✓ | | ✓ |  | ✓ | ✓ |  | ✓ |
| Damush 2011 | ✓ | | ✓ | ✓ | ✓ | ✓ |  |  |
| Damush 2016 |  | | ✓ |  |  | ✓ | ✓ | ✓ |
| Devasahayam 2014 | ✓ | | ✓ |  | ✓ | ✓ |  |  |
| Fu 2020 | ✓ | | ✓ |  |  | ✓ | ✓ |  |
| Harel-Katz 2020 | ✓ | |  | ✓ |  | ✓ |  |  |
| Harwood 2012 | ✓ | | ✓ |  |  | ✓ | ✓ |  |
| Heron 2019 |  | | ✓ |  | ✓ |  |  |  |
| Hoffmann 2015 | ✓ | | ✓ |  |  | ✓ |  | ✓ |
| Jones 2016 | ✓ | | ✓ |  |  |  | ✓ |  |
| Kalav 202 | ✓ | | ✓ | ✓ | ✓ | ✓ |  |  |
| Kendall 2007 | ✓ | | ✓ |  | ✓ | ✓ |  |  |
| Kessler 2017 | ✓ | | ✓ |  |  | ✓ |  |  |
| Lee 2023 | ✓ | | ✓ | ✓ | ✓ | ✓ | ✓ | ✓ |
| Li 2024 | ✓ | | ✓ | ✓ | ✓ | ✓ |  | ✓ |
| Lin 2022 |  | | ✓ |  | ✓ | ✓ |  | ✓ |
| Lo 2018 |  | | ✓ |  | ✓ | ✓ | ✓ | ✓ |
| Lo 2023a |  | |  |  | ✓ | ✓ | ✓ |  |
| Lo 2023b | ✓ | | ✓ | ✓ | ✓ | ✓ |  |  |
| Lund 2011 |  | | ✓ |  | ✓ | ✓ | ✓ |  |
| Mayo 2015 |  | | ✓ |  |  | ✓ | ✓ |  |
| McKenna 2015 | ✓ | | ✓ |  | ✓ | ✓ |  |  |
| Minshall 2020 | ✓ | | ✓ |  | ✓ | ✓ |  |  |
| Pallesen 2024 | ✓ | | ✓ | ✓ |  |  | ✓ |  |
| Sabariego 2013 | ✓ | | ✓ |  | ✓ | ✓ |  | ✓ |
| Sahely 2024 |  | | ✓ |  | ✓ | ✓ |  |  |
| Sakakibara 2022 |  | | ✓ |  | ✓ | ✓ |  | ✓ |
| Shaw 2020 |  | | ✓ |  | ✓ | ✓ |  |  |
| Sit 2018 |  | | ✓ |  | ✓ | ✓ | ✓ |  |
| Tielemans 2015 |  | | ✓ |  | ✓ | ✓ | ✓ | ✓ |
| Towfighi 2020 |  | | ✓ |  | ✓ | ✓ | ✓ |  |
| Tsai 2024 |  | | ✓ |  |  | ✓ |  |  |
| Visser 2014 | ✓ | | ✓ | ✓ |  |  |  |  |
| Wolf 2016 | ✓ | | ✓ | ✓ |  | ✓ | ✓ | ✓ |
| Wolf 2017 | ✓ | |  | ✓ | ✓ | ✓ |  | ✓ |
| **Total** | 26 | | 39 | 13 | 30 | 40 | 16 | 16 |

**Supplementary Table 4. Content and dose of included self-management interventions**

| **Author** | | **Targeted behaviour** | **Theoretical rational** | | **Intervention content** | **Dose and duration** |
| --- | --- | --- | --- | --- | --- | --- |
| Aben 2013 | Management of memory functioning | | | Not reported | Group-based program  Education on memory and stroke, influence of beliefs and anxiety  Coping strategies for memory deficits  Training on internal and external memory deficits  Goal setting for memory-demanding tasks  Information booklet about sessions | 18 hours over 4-5 weeks (9 x 1-hour sessions, twice/week) |
| Adamit 2023 | Daily performance | | | Social cognitive theory | Success highlighted by successful logs.  Case studies based on participants’ difficulties.  Encouragement, acknowledging efforts and progress and positive feedback.  Physiological feedback and psychoeducation | 10 hours over 10 weeks (1 hour/week) |
| Amiri 2022 | Self-management behaviours | | | Not reported | Recognising the dangers and symptoms of stroke and the benefits of behaviour change  Behavioural goal setting  Anxiety and stress management methods and skills  How to move and appropriate movement exercises  Weekly sessions conducted virtually via phone and other media | Unclear dose over 6 weeks (1 hour face-to-face + 6 unspecified weekly sessions) |
| Barker-Collo 2015 | Adherence to medication | | | Not reported | Motivational interviewing regarding behaviours for secondary stroke prevention | 2.5–3 hours over 9 months (60-90 minutes at 28 days and 30 minutes at 3,6 and 9 months) |
| Bragstad 2020 | Psychosocial wellbeing | | | Sense of coherence in life theory, narrative theory | Dialogue-based intervention to promote psychosocial wellbeing  Identity values, social network, resources and goals and problem-solving around these topics  Life after stroke, bodily changes, thoughts and experiences, daily life and emotions  How stroke affects life, coping strategies, balance in life  Stories of stroke survivors told during sessions and encouraging meaningfulness  Worksheets provided for each session | 8-12 hours over 17 weeks (60-90 minutes fortnightly) |
| Brauer 2022 | Physical activity | | | Not reported | Self-monitoring of physical activity  Short and long-term goals, action plans and coping strategies for maintaining walking behaviour.  Self-management: education, behavioural instruction, self-monitoring  Feedback on goals, problem-solving, and action and coping planning | 12 hours over 8 weeks (30 minutes x 3/week) |
| Brouwer-Goossensen 2022 | Lifestyle behaviour change | | | Not reported | Motivational interviewing  Lifestyle behaviour, motivation, and opportunities for change | 45 minutes over 12 weeks (15 minutes at 4 weeks, 8 weeks, 3 months) |
| Cadilhac 2011 | Self-management behaviours | | | Not reported | Group program: Stroke self-management, sharing stroke journey, how stroke makes them feel.  Attitudes to stroke recovery, moving towards a healthy lifestyle, leisure activities, social support, financial matters.  Working with health professionals and learning to be stroke-safe for life | 20 hours over 8 weeks (2.5 hours weekly) |
| Cadilhac 2020 | Secondary stroke prevention | | | Behaviour change theory (including social cognitive theory, information-motivational-behavioural theory and operant condition) | Behaviour change messages to support personal recovery, prevention goals and level of functional ability.  Intervention delivered via SMS or email | Unclear dose over 4 weeks (1 message daily + 1 or 2 administrative or motivational messages) |
| Chen 2019 | Self-management behaviours | | | Health empowerment theory | Individual sessions: consisted of personalised information based on health needs, advice, coaching, problem-solving and self-health monitoring, goal setting.  Small group sessions: DVD on self-management and self-care  Telephone follow-up to assess goal performance, identify barriers, provide reinforcement and empowerment, teach problem-solving | 4 hours - 4 hours 40 minutes over 6 weeks (5 x 20-minute individual sessions, 1 x 60-minute group session, 4 x 20–30-minute telephone follow-ups) |
| Damush 2011 | Secondary stroke prevention | | | Self-efficacy concept within Social cognitive theory | General education on stroke and expectations for recovery  Goal setting and feedback on goals  Education on dealing with fears, and anxiety, keeping rehab appointments, following home exercises, medication adherence, problem solving, diet modification, relaxation techniques, accessing community resources, working with health care providers and lifestyle changes  Delivered via telephone | 2 hours over 12 weeks (6 x 20-minute fortnightly telephone sessions) |
| Damush 2016 | Medication adherence for secondary stroke prevention | | | Self-efficacy concept | Education on expectations after stroke, negative/positive thinking, fears, meaningful activities, follow-up medical visits, communication with providers and caregivers  Education on physical activity to improve mood and energy, coping strategies and medication adherence.  Goal setting and behavioural contracting  Delivered via telephone | Unclear dose over 12 weeks (up to 6 fortnightly telephone calls) |
| Devasahayam 2024 | Physical activity | | | Transtheoretical model of behaviour change and social cognitive theory | Group discussions focussing on acquiring self-management skills for exercise  Discussions to identify and solve barriers to exercise, understand benefits of exercise, develop strategies to embed exercise into daily routine, report back to group challenges with home exercise program | 6 hours (1 hr/week x 6 weeks) |
| Fu 2020 | Taking charge of stroke recovery | | | Self-determination theory | Family members present if requested  Supported to identify what and who is important and priorities for the next 12 months, describe desired outcomes.  Illustrated workbook to structure priorities using headings such as mobility and activities of daily living, communication, information needs, financial issues, emotional needs, supports, and stroke prevention | 1 x 30–60-minute session: Take Charge 1  1-2 hours over 6 weeks (2 x 30–60-minute sessions 6 weeks apart): Take Charge 2 |
| Harel-Katz 2020 | Home, community, work and social participation | | | Not reported | Group program: Learning and practising self-management skills such as problem-solving and decision-making; improving participants’ self-efficacy; applying self-management skills, analysing difficulties in performing daily activities and finding strategies to improve participation  Handbook about course and relaxation CD provided | 30 hours over 12 weeks (2.5 hours/week) |
| Harwood 2012 | Self-directed rehabilitation | | | Not reported | DVD: Education about stroke and stroke recovery, overcoming adversity, personal and family roles, meaningful activity, participation, and where to access resources for people following stroke.  Take Charge Session: Personalised assessment, goal setting, education on mobility, activities of daily living, communication, emotion, information needs, finance, extended family, stress management, and secondary prevention. | 1 x 80-minute session  Participants were encouraged to view the DVD as many times as they wished |
| Heron 2019 | Lifestyle behaviour change | | | Not reported | Healthy Brain Rehabilitation Manual  UK physical activity guidelines and how to achieve the targets.  Motivational interviews to encourage goal attainment.  Provision of pedometer and daily step-count diary | Unclear dose over 9 weeks (Telephone calls at 1, 4, and 9 weeks) |
| Hoffmann 2015 | Managing depression and anxiety symptoms | | | Not reported | First two sessions covered experience of having a stroke and goal setting using the principles of motivational interviewing.  Next five sessions incorporated psychoeducation components, graded activity participation and behavioural activation, cognitive techniques, cognitive rehabilitation and skills training, and family support.  The final session covered program review, goal setting and future planning. | 8 hours over 8 weeks (1 hour/week) |
| Jones 2016 | Self-management behaviours | | | Social cognitive theory and self-efficacy | Staff trained to incorporate seven principles (problem solving, reflection, goal setting, accessing resources, self-discovery, activity, knowledge) into each therapy session to support self-management activities | Not specified. Integrated into standard rehabilitation sessions |
| Kalav 2021 | Lifestyle behaviour change and medication adherence | | | Not reported | Booklet with information about stroke, controlling modifiable risk factors, and stroke management.  Telephone interview questions examined patient’s information, beliefs, behaviours, general health status and behavioural changes regarding the risk factors; recommendations were provided when necessary.  Reminder messages related to self-management strategies  All but first session delivered via telephone | 1.5-2 hours over 8 weeks (30-45 minutes before discharge, 15-20 minute phone calls at weeks 1, 2, 4, 8 post-discharge) |
| Kendall 2007 | Psychosocial skills | | | Theories of stress and coping | Group program: Chronic Disease Self-Management (CDSM) course with stroke specific content added: healthy lifestyle, problem-solving, goal setting and communication with the healthcare team  Emphasise group support, and reinforce solution-focused behaviours | 12 hours over 6 weeks (2 hours/week) |
| Kessler 2017 | Participation | | | Social cognitive theory, goal-setting theory, solution-focussed therapy, adult learning theory | Occupational performance coaching: Goal setting, individualised education, problem-solving related to participation challenges | Unclear dose over 16 weeks (up to 10 sessions) |
| Lee 2023 | Self-management behaviours | | | Not reported | Motivational interviewing  Group-based self-management program: problem-solving, decision-making, resource utilization, self-advocacy, goal setting, and action planning.  Utilised a guided problem-solving tool, called the Activity-Barriers-Changes-Do It-Evaluation (ABCDE) framework  All sessions (except first 2 motivational interviewing sessions done while inpatient) delivered via Zoom | 11.5 hours over 6 weeks (5 x 30-minute motivational interviews, 6 x 90-minute weekly group sessions) |
| Li 2024 | Self-management behaviours and active participation | | | Social cognitive theory, person-environment-occupation-performance model | All delivery performed remotely. Education to groups, coaching to individuals via teleconferencing  Psychoeducation (teaching skills in problem-solving, decision-making, positive thinking, communication, managing symptoms, supporting participation), behavioural coaching (goalsetting) and text messaging to reinforce goals, monitoring, self-management tips | 30 hours (2.5 hr/week education, 0.5hr/week coaching for 12 weeks for 12 weeks) |
| Lin 2022 | Self-management behaviours | | | Self-efficacy theory | In-person coaching for survivors and carers before and after discharge focused on setting transitional care goals; enhancing and improving self-care and physical function; modifying the home environment, medication management; and preventing stroke adverse events.  Weekly follow-up to discuss concerns, provide resources and enhance motivation  Weekly follow-up provided via telephone | >3-4 hours over 12 weeks (2 x 20-30 minute coaching pre-discharge, dose of fortnightly follow-up sessions over 12 weeks unclear, 10-15 minutes phone call each week) |
| Lo 2018 | Self-management behaviours | | | Construct of self-efficacy and outcome expectation | Combined individual and group delivery: Based on self-efficacy constructs such as goal setting, modelling and verbal persuasion by peer stroke participants and nurse facilitator.   - Encouraged to acknowledge incremental successes, reinforce expectations of positive outcomes, and practice strategies to manage stroke - Workbook to record goals and action plans - Two DVDs with survivor stories about managing stroke successfully. | >5.5 hours over 4 weeks (1.5 hours at home; 2 x 2-hour group sessions, 3 x follow-up phone calls unspecified duration) |
| Lo 2023a | - Self-management behaviours and participation | | | - Principles of self-efficacy | - Video calls with survivor and carer: discuss recovery and self-management progress - Online platform containing 85 educational videos in 9 chapters: nutrition, exercise, medications, community services, peer-sharing, experts’ advice; and videos for enhancing caring skills and confidence, support to caregivers. - Tablet and BP monitoring device to access the website and monitor their BP - Hotline to discuss non-urgent health and psychological concerns outside session times - All consultations via video | >3-4.5 hours over 6 months (30-45 minutes/month video-call, plus 1 phone call [unspecified duration]/month)  Unlimited access to online platform |
| Lo 2023b | - Self-management behaviours | | | - Self-efficacy theory and outcome expectation | - Supported to set goals and action plans, review progress, adjust action plans, reinforcement of positive outcomes - Guided to problem-solve and decide how to sustain self-management behaviours - Resource package (available in print and online): workbook, health and life planning toolkit, access to 15 videos of peer stories, reference guide on building confidence and self-management skills. - Hotline available to contact health professional with queries about stroke Coaching sessions via phone | >6-8 hours over 8 weeks (1.5-2 hours x 4 home visits, 5 telephone coaching sessions [unspecified duration]) |
| Lund 2011 | - Lifestyle behaviour change | | | - Not reported | - Group program - Lifestyle Redesign® program: incorporating individual needs assessment, peer-exchange, self-reflection, discussions, lectures and outings | 72 hours (1 x 2 hr session/week, 36 sessions in total) over 9 months |
| Mayo 2015 | - Participation | | | - Education theory of project-based learning, cognitive-behavioural theory | - Group program - Supported to set goals and develop action plans that can be met by developing internal resources and existing community-based resources. Supported to work with group members - Exercise: aerobic exercise, core and peripheral strength, balance, flexibility, rapidity of movements | 72 hours over 3 months (2 x 3 hours/week) |
| McKenna 2015 | - Self-management behaviours | | | - Principles of self-efficacy | - One-to-one sessions to promote goal setting, monitoring of progress and problem solving. - Stroke workbook to record goals and progress, read vignettes of survivors’ experiences | 6 hours over 6 weeks (1 hour/week) |
| Minshall 2020 | - Self-management behaviours | | | - Collaborative therapy framework | - Stroke survivors and carers - Structured workbook: education (what is optimal health, I-can-do model medication; collaborative partners and strategies; timeline activities; visioning and goal setting; building health plans; my health journal), and self-management and reflective exercises - Skill building: problem solving, stress management, goal setting - Flexible delivery (face-to-face, telephone, Skype) | 9 hours over 3 months (1hour/week for 8 weeks plus one booster at 3 months) |
| Pallesen 2024 | - Self-management behaviours and activating social network | | | - Concept of self-efficacy | - Stroke survivors and carers - Interview with survivor, carer and therapist to build relationship, establish survivor’s areas of importance (home, work, activities, friends) - Activating social network so network supports survivor to work towards goal - Face-to-face support a core element. Shifted to virtual/phone during COVID lockdowns | >4.5-8 hours over 9 months (Introductory session and interview [unspecified durations], plus 6-8 supporting sessions of 45-60 mins) |
| Sabariego 2013 | - Individually selected function | | | - Social cognitive theory | - Group program - Identification of areas of functioning that are problematic post-stroke, supported to identify solutions to problems, supported to seek relevant information | 3 hours over 5 days (3 x 60 min sessions) |
| Sahely 2024 | - Physical activity | | | - Not reported | - Group and individual sessions delivered in person at home and over Zoom - Education about stroke, improving mobility, self-management and safety - Home exercise program provided - Exercise booklets, pedometer and diary for recording exercises provided - Peer advisor provided support for home exercise program | Unclear dose over 12 weeks (online group sessions every 2 weeks for 12 weeks, individual phone call alternate weeks) |
| Sakakibara 2022 | - Secondary stroke prevention | | | - Social cognitive theory, control theory | - Individual stroke coaching sessions to motivate patients to change behaviour and improve health - Self-management manual, self-monitoring kit, health report card about stroke risk factors. - Delivered via telephone | 4 hours–7 hours 50 mins over 6 months (7 x 30-60-min coaching sessions and 5 x 5-10-min ‘check-in’ calls) |
| Shaw 2020 | - Individually selected function | | | - Not reported | - Delivered to survivor +/- carer - Identification of patient’s progress, current rehabilitation needs and service provision. - Action-planning for each rehabilitation goal; verbal advice and encouragement - Signposting and referral to local activities, stroke services, community organisations or voluntary services. - Reviews conducted over telephone | Unclear duration over 18 months (reviews of unclear duration at months 1, 3, 6, 12, 18) |
| Sit 2018 | - Self-management behaviours | | | - Theory of health empowerment | - Groupwork to build partnership with peers and facilitator. Building self-efficacy (through mastery, verbal persuasion, vicarious experience and physiological feedback), developing core self-management skills, articulating goals, action planning - Individual work at home to action plans, using workbook - One face-to-face booster session to review progress, monitor or modify goals - Stroke Self-Management workbook | >3 hours over 3 months (30 min/week for 6 weeks, 1 booster session of unclear duration) |
| Tielemans 2015 | - Pro-active coping | | | - Not reported | - Group program - Proactive action planning strategies on: handling negative emotions, social relations and support, participation in society, and less visible stroke consequences - Information provision, peer group working, goal setting, action planning, monitoring progress - Workbook provided | 14 hours over 10 weeks (2-hour sessions/week for 6 weeks, 1 × 2-hour booster session) |
| Towfighi 2020 | - Lifestyle behaviour change | | | - Not reported | - Group program designed to address secondary stroke prevention - Education on lifestyle practice, peer exchange, personal exploration including goal-setting, direct experience through participation in a relevant activity - Self-management tools (pedometers, food and activity logs), binder for handouts and action plans | 12 hours over 6 weeks (2 hours/week) |
| Tsai 2024 | - Self-management behaviours | | | - Not reported | - Delivered to survivor carer dyads - Nurse-led counselling to assess family care needs - Specific Thematic Nursing Care Action for coaching. Priority care guidance provided according to needs through specific themed care action modules | 60-80 minutes over 4 weeks (2 x 30-40 min sessions) |
| Visser 2014 | - Problem-solving | | | - Not reported | - Group program - Divided problem solving into 4 steps: define problem and goal; generate multiple solutions; select a solution; and implement and evaluate. | 12 hours over 8 weeks (1.5 hours/week) |
| Wolf 2016 | - Participation | | | - Social learning theory and mechanism of self-efficacy | - Group program, problem-solving using Chronic Disease Self-Management Program and additional components specific to stroke - Skill building in problem-solving, decision making, resource utilisation, client/provider/service partnerships, action planning and self-tailoring, communicating with family and friends, community participation | Unclear duration over 12 weeks (1 session/week) |
| Wolf 2017 | - Self-management behaviours | | | - Not reported | - Group program based on Chronic Disease Self-Management Program focused on Medical management, Role management, Emotional management - Information provision, problem solving, action planning, coping strategies, decision-making | 12 hours over 6 weeks (2 hours/week) |

**Supplementary Table 5. Risk of bias of included studies**

| Author | Sequence generation | Allocation concealment | Blinding of participants/ personnel | Blinding outcome assessment | Incomplete outcome data | Selective reporting | Other sources of bias |
| --- | --- | --- | --- | --- | --- | --- | --- |
| Aben 2013 |  |  |  |  |  |  |  |
| Adamit 2023 |  |  |  |  |  |  |  |
| Amiri 2022 |  |  |  |  |  |  |  |
| Barker-Collo 2015 |  |  |  |  |  |  |  |
| Bragstad 2020 |  |  |  |  |  |  |  |
| Brauer 2022 |  |  |  |  |  |  |  |
| Brouwer-Goossensen 2022 |  |  |  |  |  |  |  |
| Cadilhac 2011 |  |  |  |  |  |  |  |
| Cadilhac 2020 |  |  |  |  |  |  |  |
| Chen 2019 |  |  |  |  |  |  |  |
| Damush 2011 |  |  |  |  |  |  |  |
| Damush 2016 |  |  |  |  |  |  |  |
| Devasahayam 2024 |  |  |  |  |  |  |  |
| Fu 2020 |  |  |  |  |  |  |  |
| Harel-Katz 2020 |  |  |  |  |  |  |  |
| Harwood 2012 |  |  |  |  |  |  |  |
| Heron 2019 |  |  |  |  |  |  |  |
| Hoffmann 2015 |  |  |  |  |  |  |  |
| Jones 2016 |  |  |  |  |  |  |  |
| Kalav 2022 |  |  |  |  |  |  |  |
| Kendall 2007 |  |  |  |  |  |  |  |
| Kessler 2017 |  |  |  |  |  |  |  |
| Lee 2023 |  |  |  |  |  |  |  |
| Li 2024 |  |  |  |  |  |  |  |
| Lin 2022 |  |  |  |  |  |  |  |
| Lo 2018 |  |  |  |  |  |  |  |
| Lo 2023a |  |  |  |  |  |  |  |
| Lo 2023b |  |  |  |  |  |  |  |
| Lund 2012 |  |  |  |  |  |  |  |
| Mayo 2015 |  |  |  |  |  |  |  |
| McKenna 2015 |  |  |  |  |  |  |  |
| Minshall 2020 |  |  |  |  |  |  |  |
| Pallesen 2024 |  |  |  |  |  |  |  |
| Sabariego 2013 |  |  |  |  |  |  |  |
| Sahely 2024 |  |  |  |  |  |  |  |
| Sakakibara 2022 |  |  |  |  |  |  |  |
| Shaw 2020 |  |  |  |  |  |  |  |
| Sit 2018 |  |  |  |  |  |  |  |
| Tielemans 2015 |  |  |  |  |  |  |  |
| Towfighi 2020 |  |  |  |  |  |  |  |
| Tsai 2024 |  |  |  |  |  |  |  |
| Visser 2014 |  |  |  |  |  |  |  |
| Wolf 2016 |  |  |  |  |  |  |  |
| Wolf 2017 |  |  |  |  |  |  |  |

Other source of bias: small sample size (<62 participants, based on sample size calculation using Stroke Self-Efficacy Scale by Kalav 2021)

| High risk of bias |  |
| --- | --- |
| Unclear risk of bias |  |
| Low risk of bias |  |

**Supplementary table 6. Meta-regression: effect of components on self-efficacy**

|  | Coefficient | P-value | 95% Confidence interval |
| --- | --- | --- | --- |
| Telehealth component | -0.34 | 0.004 | -0.56 to -1.1 |
| Group component | 0.17 | 0.20 | -0.09 to 0.44 |
| Additional resources provided | -0.23 | 0.07 | -0.49 to 0.02 |
| Intervention duration >7 hours | 0.34 | 0.005 | 0.10 to 0.57 |


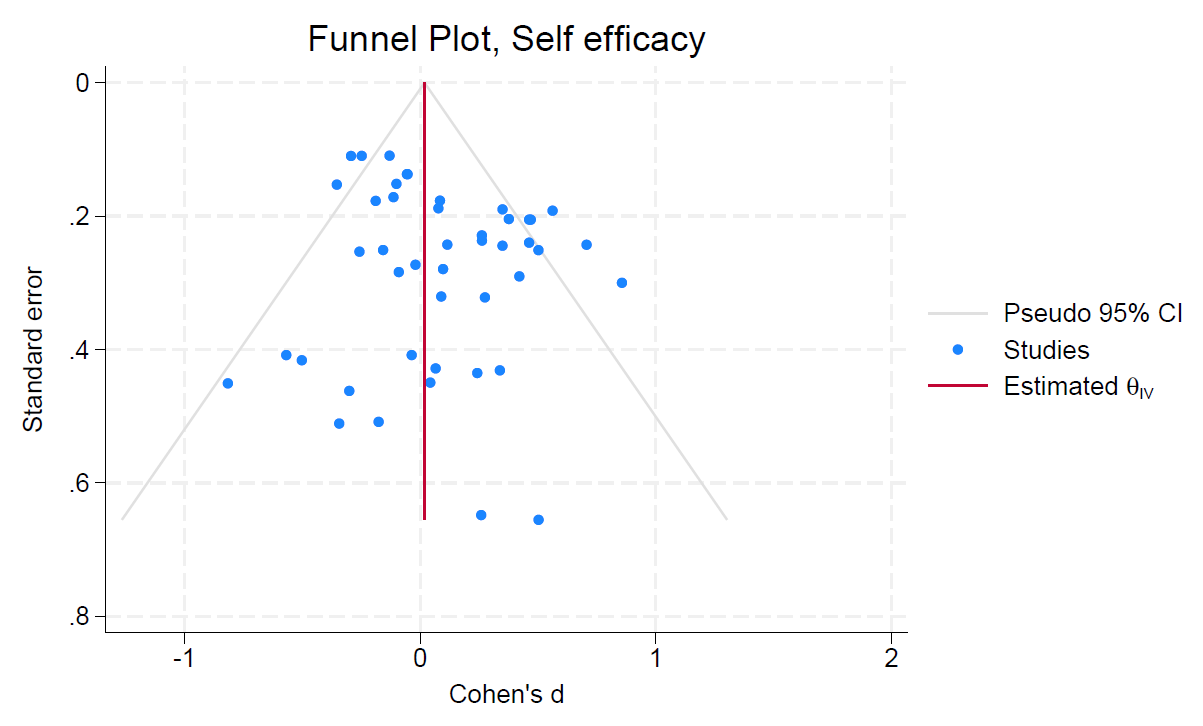


**Supplementary Figure 1. Funnel plot self-efficacy**


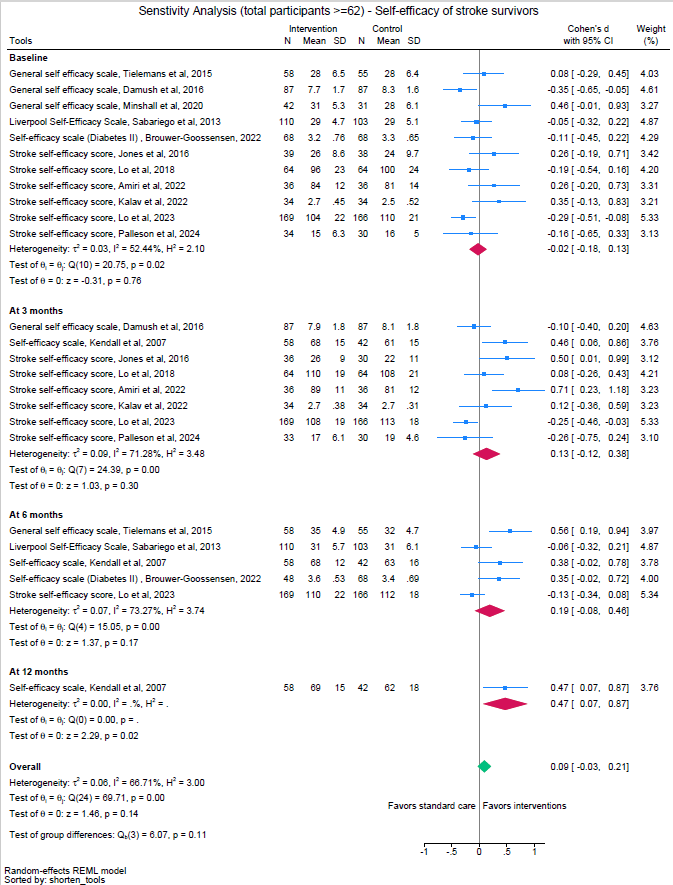


**Supplementary Figure 2. Sensitivity analysis of Self-efficacy following removal of trials with high risk of bias from more than one source**


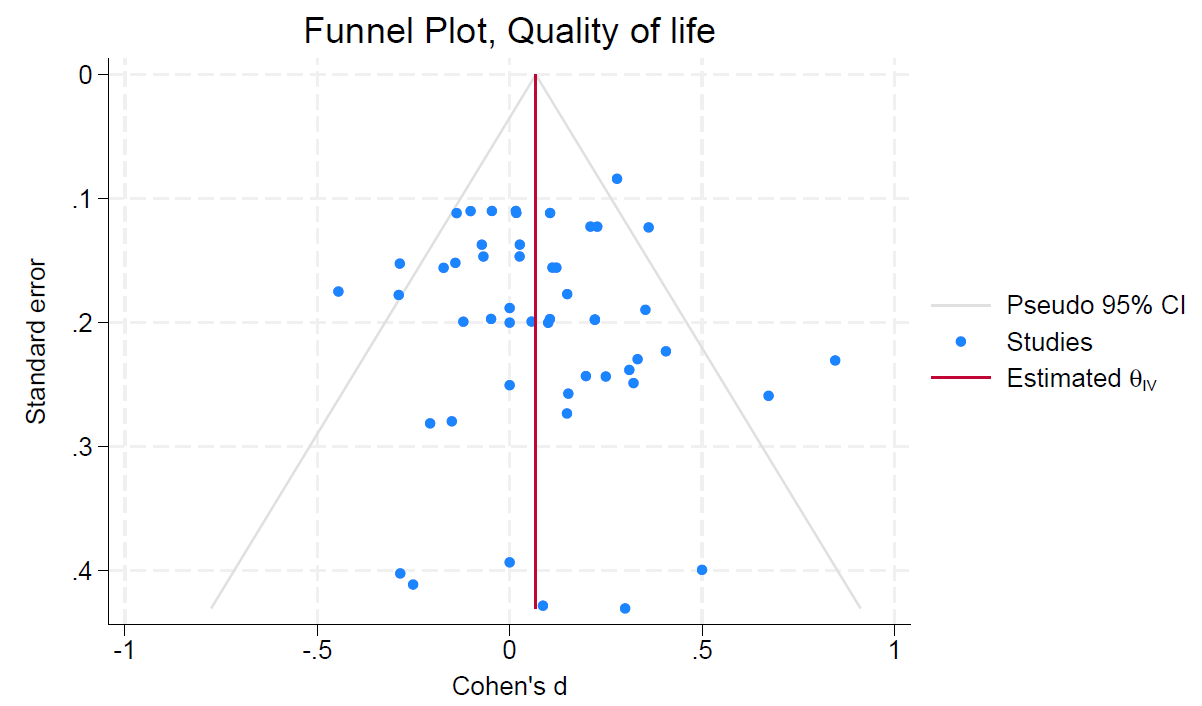


**Supplementary Figure 3. Funnel plot: Quality of life**


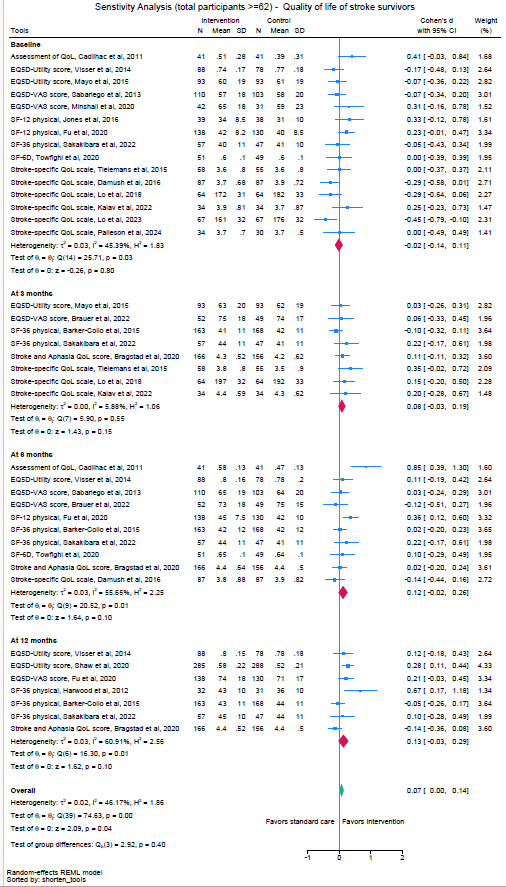


**Supplementary Figure 4. Sensitivity analysis of Health-related quality of life following removal of trials with high risk of bias from more than one source**

| **7** | | | | | | | **№ of patients** | | **Effect** | | **Certainty** | **Importance** |
| --- | --- | --- | --- | --- | --- | --- | --- | --- | --- | --- | --- | --- |
| **№ of studies** | **Study design** | **Risk of bias** | **Inconsistency** | **Indirectness** | **Imprecision** | **Other considerations**  **(Publication bias)** | **Intervention** | **Usual care** | **Relative**  **(95% CI)** | **Absolute**  **(95% CI)** |  |  |
| **Self-efficacy (3-months)** | | | | | | | | | | | | |
| 14 | Randomized trials | Not serious | Serious ^a^ | Not serious | Not serious | None ^b^ | 595 | 563 | - | SMD 0.18  (-0.03, 0.38) | Moderate | Important |
| **Health related quality of life (3-months)** | | | | | | | | | | | | |
| 13 | Randomized trials | Not serious | Not serious | Not serious | Not serious | None ^c^ | 789 | 756 | - | SMD 0.10  (0.00, 0.20) | High | Important |

a Degree of inconsistency indicated by (I2 = 58%, Q (35) = 83.23, p<0.00)

b Regression-based test of small study effects (Egger’s test: z= 0.89, p = 0.37) and symmetrical shape of Funnel plots has shown no significant effect of small studies or publication bias in the analysis

c There were no signs of significant publication bias as the test of small study effects (Egger’s test: z= 0.80, p = 0.42) and symmetrical shape of Funnel plots had no significant changes.

**Supplementary table 6.** **Meta-regression: effect of components on health-related quality of life**

|  | Coefficient | P-value | 95% Confidence interval |
| --- | --- | --- | --- |
| Telehealth component | 0.01 | 0.89 | -0.15 to 0.17 |
| Group component | 0.08 | 0.31 | -0.08 to 0.25 |
| Additional resources provided | 0.06 | 0.41 | -0.08 to 0.20 |
| Intervention duration >7 hours | -0.05 | 0.47 | -0.19 to 0.09 |
